# Control of the injection velocity of embolic agents in embolization treatment

**DOI:** 10.1186/s12938-023-01126-7

**Published:** 2023-06-15

**Authors:** Dongcheng Ren, Bo Zhou, Jiasheng Li, Shijie Guo, Baolei Guo

**Affiliations:** 1 Academy for Engineering and Technology, Fudan University, 220 Handan Rd., Shanghai, China; 2 Shanghai Engineering Research Center of AI & Robotics, 539 Handan Rd., Shanghai, 200433 China; 3 Engineering Research Center of AI & Robotics, Ministry of Education, 539 Handan Rd., Shanghai, China; 4 Department of Interventional Radiology, Fudan University Zhongshan Hospital, 180 Fenglin Rd., Shanghai, China; 5 Department of Vascular Surgery, Fudan University Zhongshan Hospital, 180 Fenglin Rd., Shanghai, 200032 China; 6 National Clinical Research Center for Interventional Medicine, 180 Fenglin Rd., Shanghai, China

**Keywords:** Biofluid dynamics, Embolization, Interventional surgery, Injection velocity, Porous media

## Abstract

**Background:**

Embolization is a common treatment method for tumor-targeting, anti-organ hyper-function, and hemostasis. However, the injection of embolic agents largely depends on the experiences of doctors, and doctors need to work in an X-ray environment that hurts their health. Even for a well-trained doctor, complications such as ectopic embolism caused by excessive embolic agents are always inevitable.

**Results:**

This paper established a flow control curve model for embolic injection based on local arterial pressure. The end-vessel network was simplified as a porous media. The hemodynamic changes at different injection velocities and embolization degrees were simulated and analyzed. Sponge, a typical porous medium, was used to simulate the blocking and accumulation of embolic agents by capillary networks in the in vitro experimental platform.

**Conclusions:**

The simulation and experimental results show that the local arterial pressure is closely related to the critical injection velocity of the embolic agent reflux at a certain degree of embolization. The feasibility of this method for an automatic embolic injection system is discussed. It is concluded that the model of the flow control curve of embolic injection can effectively reduce the risk of ectopic embolism and shorten the time of embolic injection. The clinical application of this model is of great value in reducing radiation exposure and improving the success rate of interventional embolization.

## Introduction

Interventional embolization has been widely used in clinical practice. Embolization of tumor tissue can be performed by occluding blood vessels, rendering the tissue ischemic and necrotic. Embolic particles can serve as carriers completing the targeted delivery of molecules such as drugs and genes [1]. For instance, transcatheter arterial chemoembolization (TACE) is widely acknowledged as a frequently employed non-surgical treatment for HCC, leading to significant survival improvements in patients with liver cancer [2]. TACE offers several advantages over traditional surgery, including shorter hospitalization, minimal trauma, and a higher overall survival rate [3]. Interventional embolization can employ a variety of embolic agents, such as metal coils, liquid or gel agents, and particulate agents. Each type of embolic agent has its advantages and disadvantages and is suitable for specific clinical scenarios [4]. Currently, particulate embolic agents are the mainstay for transarterial embolization (TAE) or chemoembolization (TACE) of liver tumors, especially hepatocellular carcinoma (HCC) [5]. But the non-target embolization and the ischemia caused by the embolization of the vital area may be considered the main limits of the embolization [6]. This paper mainly focuses on the analysis, simulation, and in vitro experimental study of hemodynamic changes caused by particle embolic agents injected into target vessels.

There is always a chance that an embolic agent can lodge in the wrong place and deprive normal tissue of its oxygen supply, that is, ectopic embolization [7]. Placing the (micro) catheter in a precise position is a means to prevent damage to normal tissue. However, more important is the fine control of embolic agent injection. Injection under continuous fluoroscopy is the main means of reducing the risk of non-target embolization in current surgery [8]. The endpoint of embolization is determined by digital subtraction angiography (DSA) to avoid excessive injection of the embolic agent.

The consensus statement from the international expert panel of the International Society of Multidisciplinary Interventional Oncology (ISMIO) has clearly defined the endpoint for transcatheter arterial chemoembolization in hepatocellular carcinoma. The endpoint of TACE is defined at angiography as the presence of flow stasis of the tumor feeding arteries under fluoroscopic monitoring. Complete stasis is visualized as a static contrast column for at least 5 heartbeats. For cTACE, the optimal complete embolization is featured by the appearance of the lip iodol filling of in periphery portal veins around the tumor. For DEB-TACE, injection must be stopped once stasis is observed in the tumor feeding arteries to avoid reflux of embolic materials [9]. The clinical practice guidelines for transcatheter arterial chemoembolization (TACE) in the treatment of hepatocellular carcinoma in China (2021 version) point out that during d-TACE procedures, it is recommended to inject drug-loaded microspheres at a speed of 1 ml/min, ensuring their even distribution and suspension throughout the injection process. Suppose the flow rate of drug-loaded microspheres and contrast medium suspension in the tumor-feeding artery does not empty within 3–4 cardiac cycles. In that case, it can be considered the embolization endpoint, and bolus injection should be discontinued. Angiography has performed again after stopping for 5–15 min. If there was still tumor-staining, embolization was continued until the endpoint was reached (tumor staining disappeared) [10]. This causes not only radiation damage to doctors, but also this visual prompt is subjective and cannot accurately represent the physiological endpoint of the operation [11]. Therefore, considerable research is devoted to judging the embolic endpoint by more effective means, such as quantitative digital subtraction angiography (qDSA) technique [12]. Local arterial blood pressure was proposed as a criterion for assessing the end point of embolization in a previous study by the authors to avoid the risk of over-injection, which was confirmed in vitro and in vivo experiments [13]. However, ectopic embolization is not only due to the inaccurate determination of the endpoint of embolization, resulting in the excessive total amount of embolic agent injected.

The leading cause of ectopic embolism is the reflux of the embolic agent along the blood vessel. Too high injection velocity is also a possible factor causing embolic agent reflux [14]. Although relevant studies have proposed blocking the proximal blood vessels by physical methods to prevent ectopic embolism (anti-reflux catheter system) [15], this also hinders the blood flow and makes the embolic agent lose the driving force to move forward. The primary process of interventional embolization is that the embolic agent enters the vascular network of the lesion with blood flow. With the accumulation of embolic agents in the blood vessels, the blood vessels become blocked, and the diseased tissue loses its blood supply. Hemodynamic parameters such as blood flow, blood pressure, and flow resistance change with the injection of embolic agents. This means that the flow velocity of embolic agent injection should be adjusted with the degree of embolization to ensure proper and accurate embolic delivery to the lesion.

Embolic agent particles are generally 100–700 μm, with certain compression deformation [5]. Therefore, embolized vessels have small diameters and complex spatial structures. Whether it is a geometric model for computational fluid dynamics simulation or a structural expression in an in vitro experimental platform, it is difficult to reproduce the actual shape of blood vessels. To build an in vitro experimental platform that can characterize the main characteristics of interventional embolization and has engineering value, an effective embolization site hydrodynamic model must be established. The Winkessel model is widely used to estimate end-of-vessel resistance [16]. However, it is difficult to obtain its experimental parameters, and it is difficult to reproduce the model in vitro. Hence, the essential aspect of achieving automated injection of the embolic agent is to (1) establish a fluid dynamics model of the embolic site, (2) construct an in vitro experimental platform capable of capturing the principal features of interventional embolic surgery and possessing engineering significance, (3) analyze the variations in hemodynamic parameters during interventional embolic surgery, and (4) ascertain the target injection flow rate without embolic agent reflux at different degrees of embolization.

## Results

### Numerical simulations

**Fig. 1:**
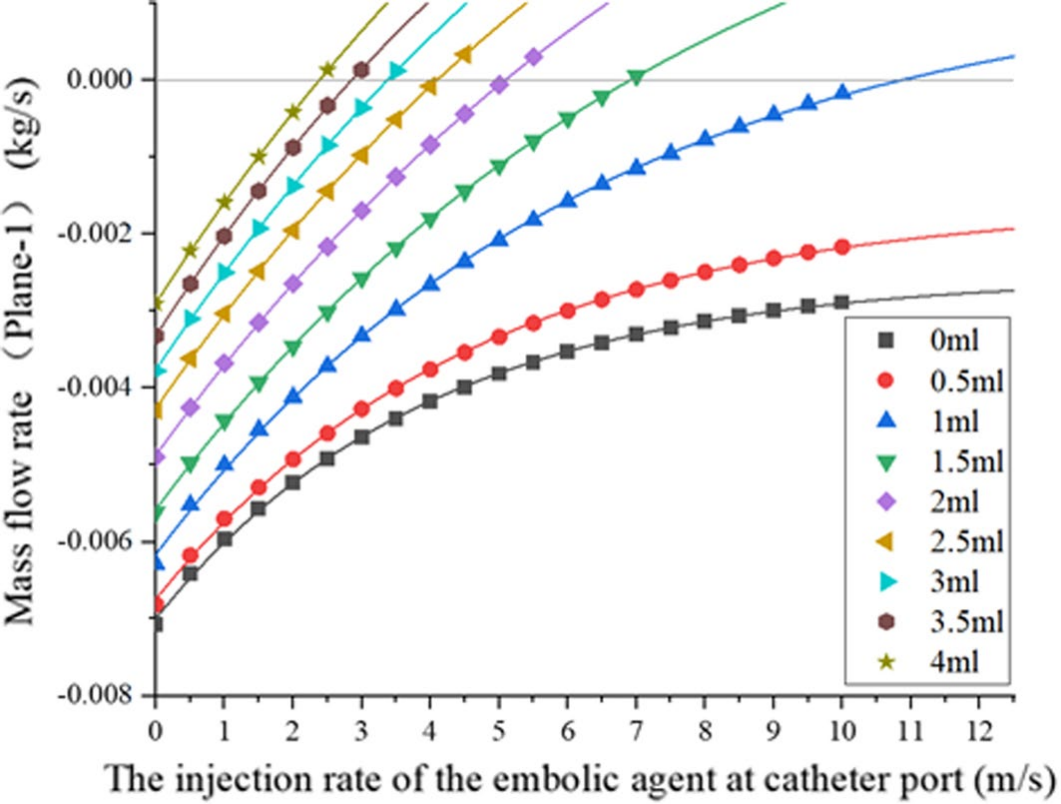
The flow of Plane1 during embolic agent injection at different injection velocities

Figure 1 shows the flow statistics of plane1 during embolic injection obtained by simulation. Negative flow indicates that the direction of blood flow is from the aorta to the end tissues. When it changes from negative to positive, the movement of blood flow changes. That is, the phenomenon of reflux of embolic agents occurs. Data points represent the results of each simulation. The critical velocity at which the flow is 0 is obtained by fitting data points of the same series into curves. Since the injection velocity of the embolic agent should not be too high, the maximum injection velocity in the simulation is 10 m/s.

### Experimental results

**Fig. 2:**
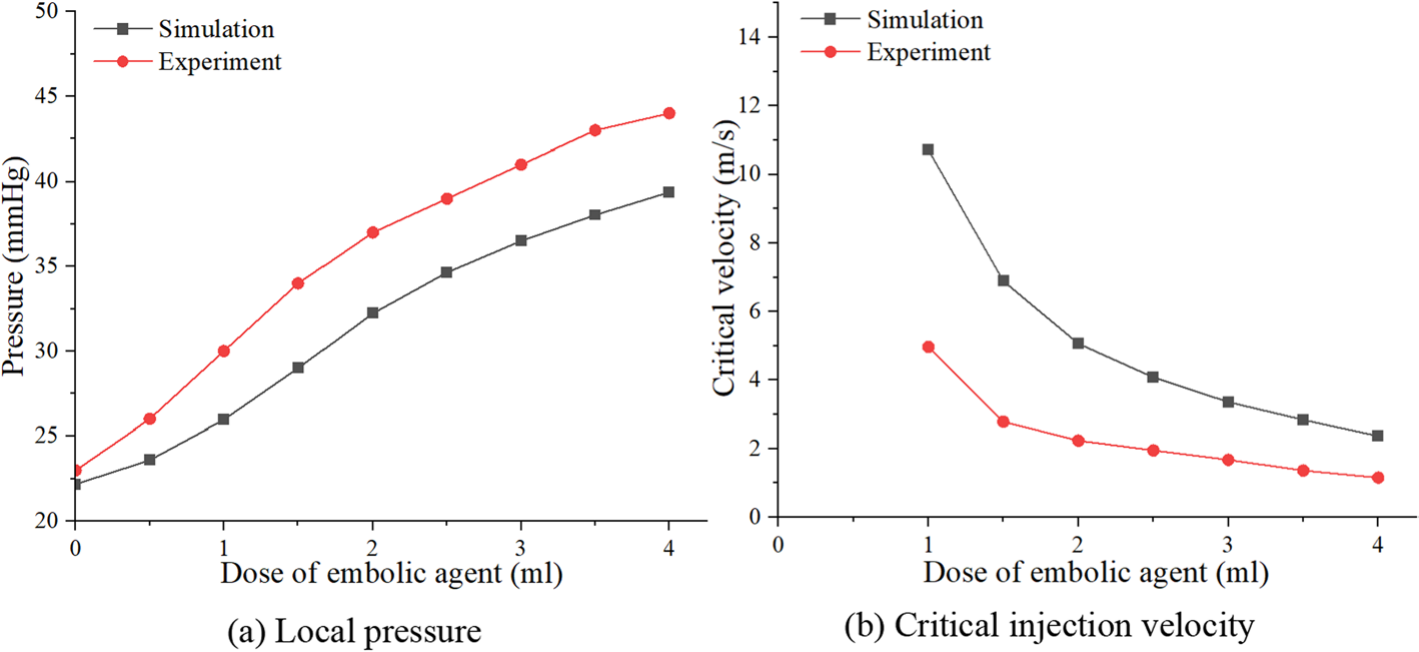
Local arterial pressure and critical velocity in simulation and experiments

**Fig. 3:**
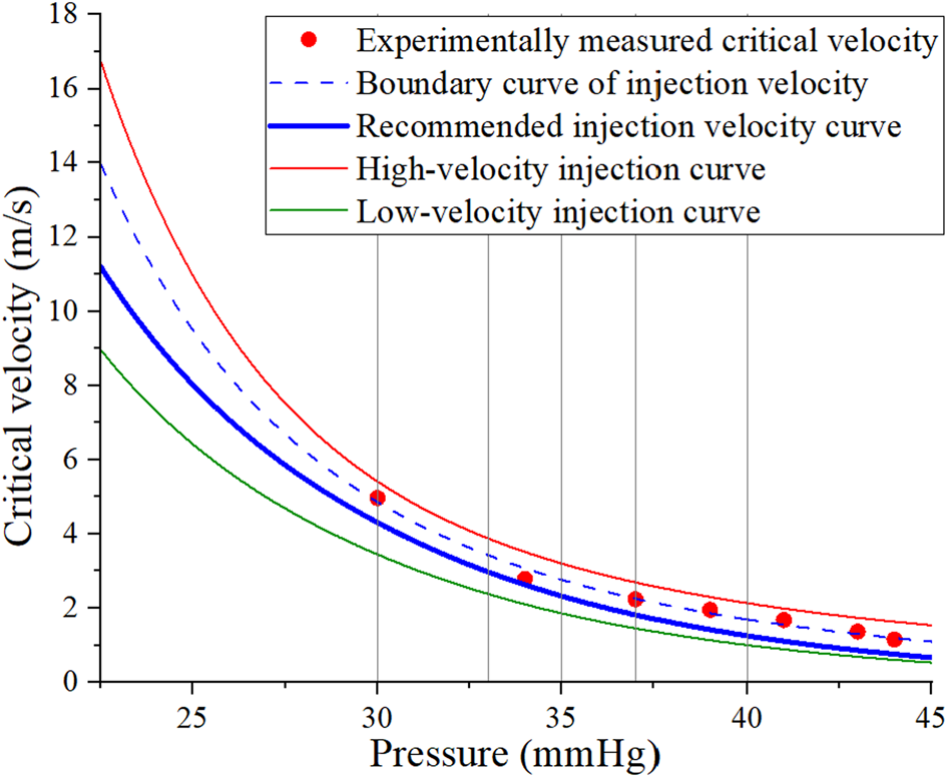
Pressure–velocity curve in the validation experiment

Figure 2 shows the relationship between embolic dose, critical injection velocity at reflux, and local pressure during embolization. The pressure is measured at point G in simulation and in vitro experiments. The injection pump can provide a maximum injection velocity of 5.58 m/s at the catheter ostia. Reflux did not occur at the top injection velocity without embolic and 0.5 ml embolic agents. Therefore, the critical velocity of reflux in both states is not recorded. Both simulation and experiment confirm that local pressure is closely related to the dose of embolic agent injected, which indicates that different degrees of embolization can be judged by measuring local pressure. It can also be found that the dose of the injected embolic agent increases, and the critical velocity for the occurrence of embolic reflux decreases. This means that the critical injection velocity at the current degree of embolization can be predicted by monitoring the local arterial pressure. Therefore, we propose the following, as shown in Fig. 3, a flow control curve model for embolic injection based on local arterial pressure.

To ensure that there is no backflow of embolic particles during embolic agent injection, the actual injection flow velocity should be located below the boundary curve. At the same time, in order to shorten the operation time and reduce radiation injury, a larger injection velocity should be selected in the area without reflux. Under this principle, we give the recommended injection velocity curve. To verify whether the injection under the control of this curve can complete the embolization quickly and without reflux, two comparison curves were set up. The velocity values of the high-velocity injection curve are higher than the recommended injection velocity curve. The velocity values of the low-velocity injection curve are lower than the recommended injection velocity curve.

Table 1 shows the experimental results with three curves as the reference of injection velocity under different degrees of embolization. The results showed that the injection of the embolic agent above the recommended injection velocity would cause obvious reflux. The recommended injection velocity and lower injection velocity will not cause reflux. This indicates a maximum allowable injection velocity for each degree of embolism during the embolic injection process, and the velocity decreases with the increase of embolization degree. At a certain degree of embolization, injections of embolic agents beyond this critical velocity are not allowed. Otherwise, a regurgitation of the embolic agent will occur. That is to say. A one-to-one relationship exists between local pressure, embolization degree, and critical velocity. Monitoring local pressure during injection will effectively avoid ectopic embolism due to excessive total dose of embolic agent (misjudgment of the endpoint of embolization) and excessive injection velocity (during embolization). At the same time, it should be noted that the lower the injection velocity, then it takes longer to reach the endpoint of embolization. Therefore, the injection of the embolic agent according to the recommended velocity curve is an effective way to complete embolization safely and efficiently.

**Table 1:** Results of validation experiment

Pressure-Point G (mmHg)	Reference curve	Injection velocity (m/s)	Reflux
30	Low-velocity injection curve	3.44	No
	Conventional injection velocity curve	4.30	No
	High-velocity injection curve	5.41	Yes
33	Low-velocity injection curve	2.38	No
	Conventional injection velocity curve	2.97	No
	High-velocity injection curve	3.86	Yes
35	Low-velocity injection curve	1.86	No
	Conventional injection velocity curve	2.32	No
	High-velocity injection curve	3.20	Yes
37	Low-velocity injection curve	1.45	No
	Conventional injection velocity curve	1.81	No
	High-velocity injection curve	2.69	Yes
40	Low-velocity injection curve	1.00	No
	Conventional injection velocity curve	1.25	No
	High-velocity injection curve	2.13	Yes

## Discussion

This paper modelled organ capillaries using porous media, and an in vitro experimental platform was established. The simulation and experimental results show that the injection velocity of the embolic agent is one of the determinants of reflux. The degree of embolization corresponds to the corresponding local pressure and a critical injection velocity for the regurgitation of the embolic agent. As the degree of embolization increases, the local pressure increases, and the critical injection velocity for reflux decreases. This demonstrates that the injection rate should decrease with the degree of embolization during the embolization process to ensure that the embolic particles do not reflux to other normal vessels during the embolization process. At the same time, monitoring local pressure can provide a new criterion for determining the degree of embolism for doctors and recommend an appropriate injection velocity based on the corresponding velocity–pressure curve. This is important for increasing the safety of interventional embolization. Of course, more in vivo experimental data are needed for the boundary curves of injection velocities for different individuals and tissues in clinical applications.

The judgment of embolization degree based on local pressure and the control of embolic injection velocity also provides a theoretical basis for the mechanization and automation of embolic injection. While some mechanized injection methods have gained wide recognition and clinical application, most of them are limited to intravenous or subcutaneous administration [17, 18]. Furthermore, the control system for mechanized liquid medicine injection relies primarily on the doctor's predetermined drug dose and injection velocity. However, due to the absence of sufficient feedback information, even though manual injection is no longer required for specific scenarios that necessitate close monitoring of drug effects, such as anesthesia or blood pressure control [19], doctors still need to continuously monitor relevant physiological indicators and provide manual intervention to the injection system when necessary. Hence, there is a more excellent application value and market demand for automatic drug injection systems with closed-loop feedback control.

Drugs are typically administered through the interventional catheter in interventional vascular embolization. Compared to intravenous injection, there is a significant increase in resistance, and the distribution of drugs after injection is greatly influenced by blood pressure and blood flow pulsation [20]. The continuous accumulation of embolic agents in the capillaries leads to complex changes in hemodynamic parameters. Some clinical practice guidelines provide only the recommended average injection velocity of the embolic agent, without specifying the details of the entire injection process. The recommended injection velocity is primarily determined by doctors based on their clinical experience, without relying on quantifiable hemodynamic parameters as guidance [2].

Some research has conducted relevant theoretical and experimental research on drug injection in vascular interventions. For instance, Qiulin et al. established a three-dimensional bifurcation arterial hemodynamic model to simulate and analyze the principles of pulsating blood flow and local hemodynamic phenomena [21]. The study discussed the influence of various injection parameters on drug solution distribution during the discontinuous injection. Lastly, the experiment involved mechanical drug injection during a specific slow ejection phase based on comparing arterial blood pressure and the predicted time phase of the cardiac cycle. However, the practical implications of the time variability of heartbeat and blood pressure on the actual injection effect have not been explored theoretically nor applied in clinical practice. Gowda et al. utilized an extracorporeal silicone vascular model to establish a correlation between the measured blood pressure values and the injected volume of embolic particles, as well as to explore the relationship among blood flow, intravascular pressure, and injection pressure [11]. However, their discussion was limited to the feasibility of blood pressure as an endpoint for embolization, and they did not extensively investigate the control of embolic agent injection velocity based on local arterial pressure. Periyasamy et al. proposed the use of quantitative digital subtraction angiography (QDSA) technology to assess the alterations in hepatic artery blood flow velocity during embolization [12]. They demonstrated that QDSA could quantify the embolization endpoint by measuring blood flow velocity in vivo in pigs. The reduction in velocity exhibited a linear correlation with the embolic dose. Undoubtedly, this offers a novel concept for regulating the injection velocity of embolic agents. However, the operational process necessitates the determination of the optimal projection angle and the acquisition of additional intraoperative DSA images, which may lead to increased radiation exposure to both doctors and patients.

The blood vessels have small diameters, and the operating environment is complex. The operation site still lacks sufficient means and methods for detecting deep intravascular-related parameters, resulting in a missing critical information feedback loop for closed-loop control in the automatic control system. Consequently, there are limited reports on applying a closed-loop control automated injection system in interventional embolization. In current interventional embolization procedures, only contrast agent injection is automated [22]. Doctors can complete the injection in the operating room by setting the contrast agent dose and the injection pressure. Because of the high risk of operation, the real-time and safety of the control system are highly required. The conventional timing or quantitative injection mode cannot meet the requirements of embolic injection. In the previous study, the authors employed local arterial pressure as the criterion to assess the endpoint of embolization, which was validated through an experimental procedure involving pig renal artery embolization. Animal experiments elucidated a technique for acquiring local arterial pressure via bilateral femoral artery puncture [13]. While these experiments were conducted in the renal artery of pigs and cannot fully replicate the conditions of tumors in the liver and other organs, they still provide valuable references for animal experiments in various organs and obtaining local arterial pressure in clinical settings. Using local blood pressure as the control parameter will undoubtedly significantly improve the safety and feasibility of automatic embolic injection. This would make up for the current shortage of medical professionals relying solely on imaging information to determine the embolization endpoint. Of course, this study ignores human blood flow pulsation characteristics, so selecting the injection flow rate may require maintaining the corresponding safety threshold with the flow rate boundary curve, which requires further investigation.

## Conclusions

This study demonstrates that measuring local blood pressure can assist physicians in determining the degree of embolism and predicting the embolic agent's non-reflux critical injection velocity. The method of determining the injection velocity of the embolic agent according to the critical velocity curve can effectively avoid reflux and realize rapid embolic agent injection. It provides a basis for the design of an embolic agent injection robot.

## Methods

In this paper, the embolic site was simulated using porous media. The simulation was conducted to analyze the hemodynamic alterations at various injection velocities and degrees of embolization. The simulation and experimental results indicate that the injection velocity of the embolic agent should decrease gradually with the increasing degree of embolization, and the appropriate non-reflux injection velocity can be determined by measuring the local pressure. Employing the recommended injection flow velocity curve for embolic agent administration can effectively mitigate the risk of ectopic embolism. Moreover, this finding lays the groundwork for developing an automated injection control system for the embolic agent.

### Simulation

**Fig. 4:**
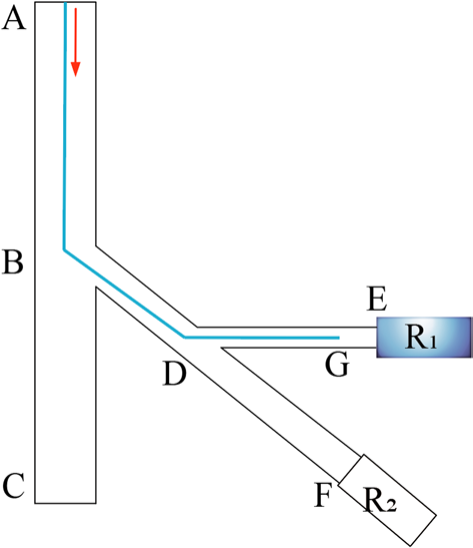
Schematic of the unit vessel model

The authors have proposed a vascular unit model in previous studies, as shown in Fig. 4 [13]. The model assumes that the target vessel is the first branch of an organ's artery. The model contains target vessel branches, other branches at the same level as the target vessel, organ arteries, and aortas. Treat all downstream vessels of the target vessel as porous media. This simplified analysis method can avoid the modeling of terminal vessels. Moreover, the resistance effect of embolism on the whole blood flow can be expressed by the momentum loss in the porous media domain [23]:

1 $${S}_{i}=-\left\{\frac{\mu }{\alpha }{v}_{i}+{C}_{2}\frac{1}{2}\rho \left|v\right|{v}_{i}\right\}$$

where *S*_*i*_ is momentum decay, *v*_*i*_ and |*v*| represent the blood flow velocity and its absolute value in different directions respectively. 1/α indicates the coefficient of viscous resistance. *μ* indicates blood viscosity. *ρ* indicates blood density. *C*_2_ is the inertial resistance coefficient.

**Fig. 5:**
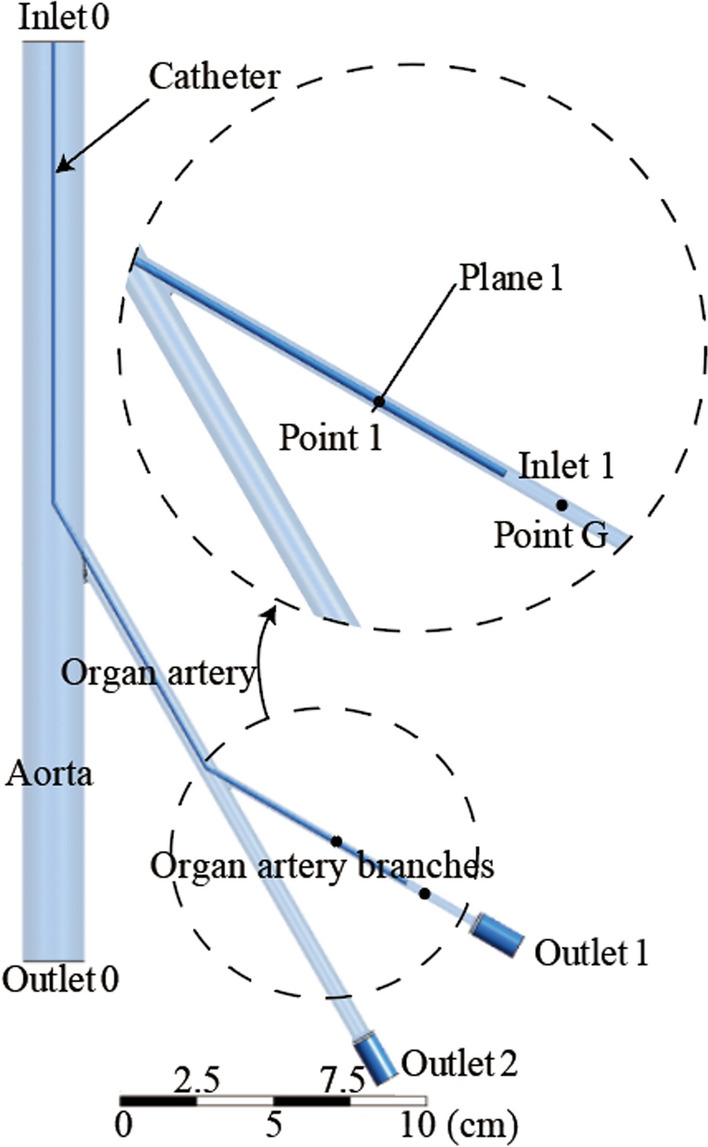
Simulation model and settings

The simulation model shown in Fig. 5 is constructed, which corresponds to the unit vessel model. The model includes the aorta, organ artery, and organ artery branches. There is an isotropic homogeneous porous media domain at the end of the organ artery to characterize the terminal resistance vessels. The relative sizes of the model were set up using the human renal vascular system as a reference in the simulation [24–27]. The diameter of the aorta is 20 mm, the diameter of the organ artery is 6 mm, and the branch of the organ artery is 3 mm. These vessel diameter settings are close to the actual vessel diameter. The hollow portion represents the vascular space occupied by the 4Fr catheter as it enters the arterial branches of the organ. The inlet, outlet, and data detection points in the simulation model are indicated in Fig. 5. The location of the monitoring points corresponds to the location of the pressure measurement points in the in vitro experimental device.

The Computational fluid dynamics (CFD) simulation meshes are created using commercial software ANSYS Mesh (ANSYS, Inc., Pennsylvanian, USA). The Fluent solver is applied for the implementation. Some model meshes are optimized for simulation accuracy and efficiency. The mesh element size is 0.2 mm for the organs' arterial branches. Since the diameter of the catheter is only about 1.3 mm, its grid element size at its wall surface is set to 0.1 mm. For larger diameter abdominal aorta and organ arteries, the mesh element sizes were 2 mm and 1 mm, respectively. The mesh element size of the end porous media domain is 0.5 mm. For lower-quality meshes such as vascular bifurcations, use the improved grid quality function in the FLUENT software to elevate the grid's Minimum Orthogonal Quality to 0.427. The total number of elements is 6.75 million. Assume laminar flow and fluid set to water. Water was used to simulate blood because blood viscosity is comparable to that of water at the arteriole level. This assumption was derived from the Fahraeus Lindqvist effect [28]. Steady-state simulation is performed, where blood flow pulsing is not considered. Moreover, arterial walls are assumed to be rigid and impermeable, and a no-slip condition is applied. The inlet 0 is set to a constant-flow inlet of 0.159 m/s. This is consistent with the flow rate provided by the in vitro experimental device. The setting facilitates the comparison and analysis of the simulation and experiment results. Outlet 0 is set to a constant-pressure outlet of 50 mmHg. The end of the porous media represents the flow of blood into the venous system, so outlet 1 and outlet 2 are set to 0 mmHg.

Continuity residuals are reduced to less than 10^–3^ at 500 iteration steps. The residual curve tends to be flat thence as the pressure values at the monitoring points tend to stabilize. Therefore, the simulation convergence threshold is set to 10^−3^. The maximum number of iterations is set to 500.

Subsequently, the blood flow parameters are simulated at different injection velocities for different degrees of embolization.

Step 1. The degree of embolization is defined by setting different porous media coefficients (Table 2). These coefficients were obtained by measuring local arterial pressure and outlet flow after injection of 0.5 ml, 1.5 ml, 2 ml, 2.5 ml, 3.5 ml, and 4 ml embolic agents in an in vitro experimental device [13]. Plane1 represents one radial section of the vessel upstream of the catheter ostia. Figure 1 shows flow through Plane 1 at different embolization degrees. It can be seen that the flow through plane 1 is a negative value without embolic agent injection. Negative numbers represent the direction of discharge from the inlet to the outlet. After embolization, the flow through the plane1 gradually approaches 0 and eventually becomes positive. This is due to reduced blood flow and eventual reflux after the end of the vessel is blocked. Therefore, the flow through Plane 1 can be used to determine whether reflux.

**Table 2:** Equivalent resistance coefficients at different embolic doses

Dose of embolic agent (ml)	Inertial resistance coefficient (10^3^/m)	Viscous resistance coefficient (10^8^/m^2^)
0	9.36	3.19
0.5	10.96	3.25
1	16.18	3.31
1.5	21.78	3.35
2	26.70	6.46
2.5	31.85	10.06
3	37.21	13.81
3.5	41.31	18.74
4	43.75	25.09
4.5	46.36	32.38
5	49.73	37.75

Step 2. Intravascular injection of embolic agents from inlet 1 results in changes in local arterial pressure and flow through plane 1. The embolic agent injection velocity is initially set to 0 for simulation. The next simulation will be performed after each 0.5 m/s increase in injection velocity. This way, the flows through plane 1 at different injection velocities and embolization degrees are obtained.

Step 3. For a specific degree of embolism, several Plane1 flow data points are obtained at different injection velocities. Fitting these data points gives the injection velocity at flow 0 (Fig. 2b). This is defined as the critical injection velocity. At the current degree of embolism, reflux occurs when the embolic agent is injected beyond this velocity.

### In vitro* experiment*

#### In vitro experimental platform

**Fig. 6:**
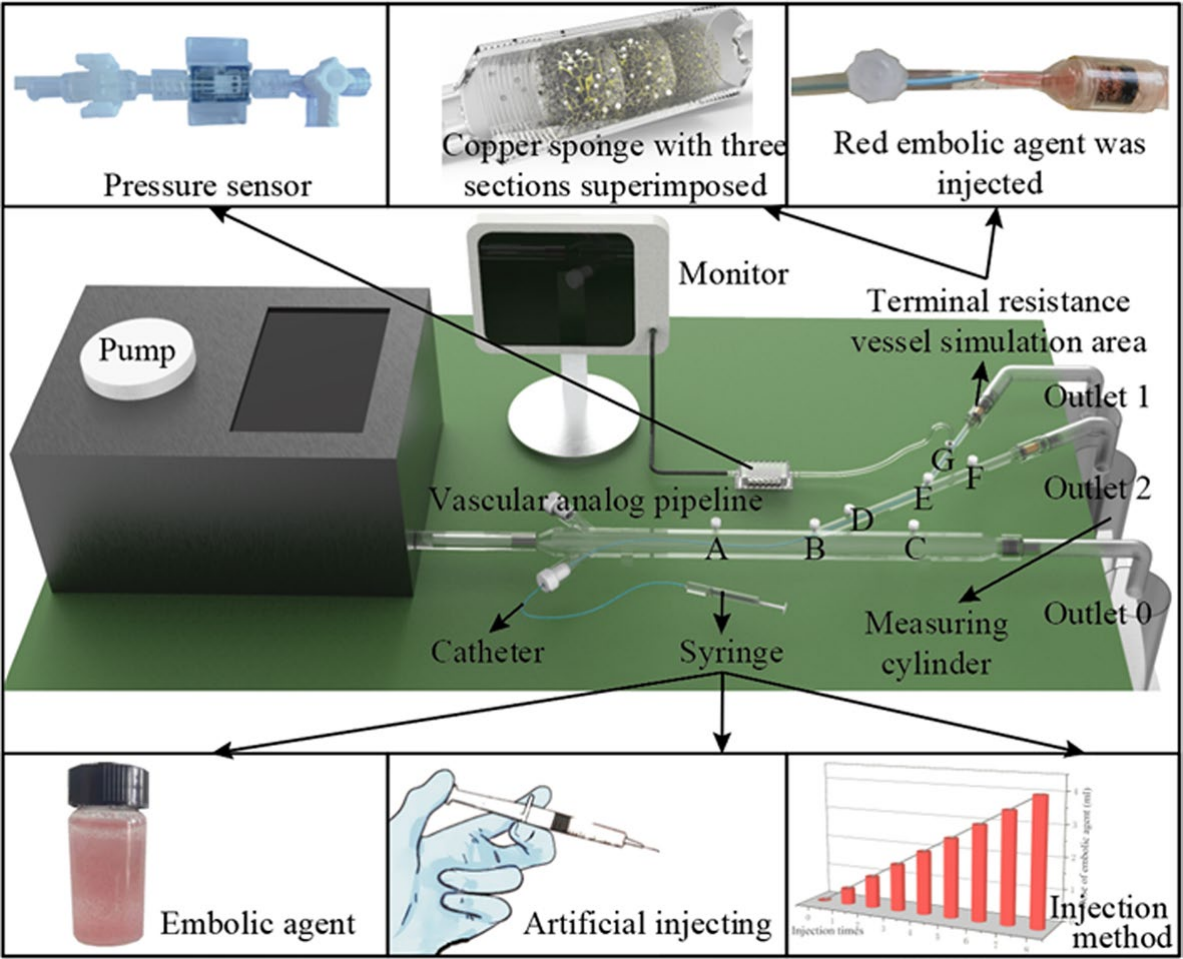
In vitro experiment platform

The in vitro experimental device, as shown in Fig. 6, is designed. The experimental device consists of a constant-flow pump, an analog vascular pipeline, terminal resistance vessel analog areas, a pressure sensor, a monitor, and other pipelines. The experimental fluid is water, and a constant flow input of 50 ml/s is provided to the experimental device through a constant flow pump (Ningbo Chuangdao 3D Medical Technology Co., Ltd.). The embolic particles used in the experiment are plastic microspheres produced by Zhiyi Microsphere Technology Co., Ltd. Three microspheres with particle sizes of 200 μm, 300 μm and 400 μm were mixed to 1 g and then injected into 20 ml brine of similar density. The microspheres are suspended in solution. This helps control the dose of the embolic agent during the experiment. The brine contains red dye to observe the flow of the embolic agent into the pipeline. The embolic agent is injected into the target vessel through a 4Fr catheter.

The vascular simulation pipeline is made of resin 3D printing and is transparent. Its structure corresponds to the unit vessel model, including interventional channels, aorta, organ artery, and organ artery branches. A plurality of quick-change interfaces connected with the pressure transducer (Abbott 42584-05, ICU Medical, Inc., California, USA) is set on the vascular simulation pipeline to measure the pressure in the pipeline. There are terminal resistance vessel analog areas at the end of the analog vascular pipeline.

Terminal resistance vessel analog areas should have the function of intercepting embolic particles and gradually occluding the accumulation of embolic particles to simulate the actual embolic process. Therefore, a variable-path channel is needed for the flow of embolic agents. On the other hand, this part corresponds to the porous media in the simulation. For this reason, a structure with three segments of copper sponge (average pore size is 0.4 mm, 0.3 mm, and 0.2 mm in flow direction) is proposed. Sponges are typical porous media. Copper sponges are easy to obtain and have a stable structure. Three different average pore sizes of sponges are stacked one by one to form a variable-path channel for the flow of embolic agents. After the embolic particles enter the sponge, they cannot flow out of the last segment because the average pore size of the sponge decreases gradually. Finally, embolic particles accumulate continuously in the sponge to simulate endovascular embolism.

#### Pressure–critical velocity curve

Embolic agents were injected into the branches of organ arteries through a 4Fr vertebral catheter, 0.5 ml at a time. The flow of the red embolic agent in the blood vessels was observed during the injection to confirm that the embolic agent did not reflux to other vessels. After each injection, the remaining embolic particles in the catheter are flushed into the branch vessels with water.

**Fig. 7:**
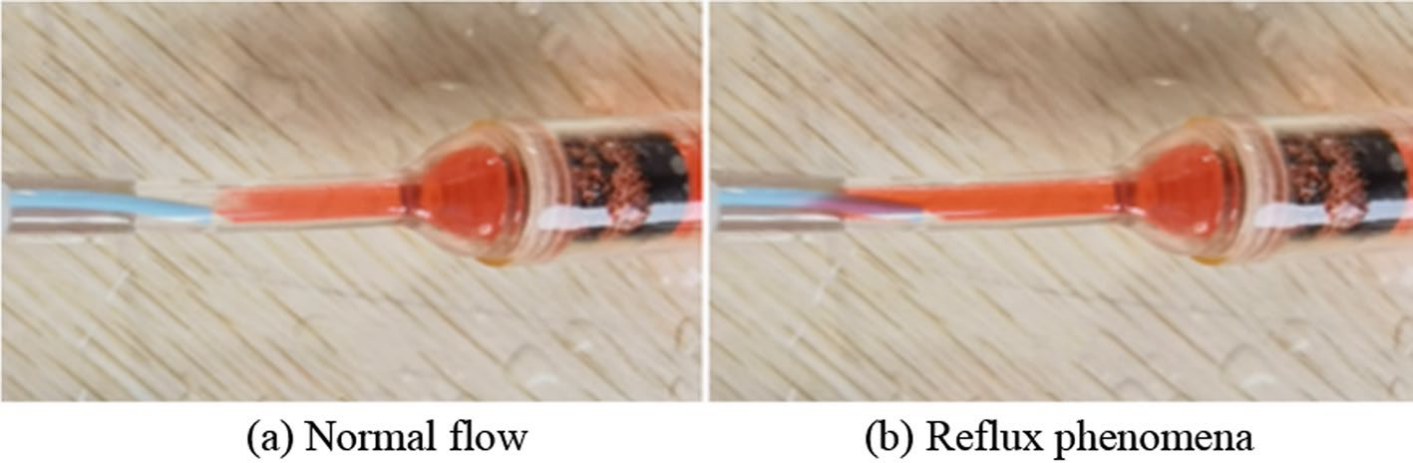
Normal flow and reflux phenomena in the experiment

The embolic agent was then replaced with water mixed with a red dye. It is injected into the branch vessels at a constant velocity using an auto-injection pump. Observe the flow of red fluid through the pipeline to determine whether reflux has occurred. In Fig. 7a, normal flow is shown. Reflux is judged to have occurred if the red liquid exceeds the white area on the head of the catheter, as shown in Fig. 7b. If reflux occurs, the injection rate will be reduced. If not, the injection rate will be increased, and the observation will continue until the critical velocity at which reflux does not occur at the degree of embolism is found (Fig. 2b). Blood pressure within the embolic branch is also recorded (Fig. 2a). There is a strong correlation between local blood pressure, embolization degree, and critical injection velocity. By fitting these data points, embolization flow control curves based on local arterial pressure are obtained in vitro (Fig. 3).

#### Validation experiment

This experiment aims to verify whether the above flow control curves for injection based on local arterial pressure effectively control embolic agent reflux. Five embolization degrees (five pressure values) are selected for validation within the effective pressure range.

Step 1. Replace the copper sponge the in the vitro device. Inject the embolic agent. During the injection, keep the injection at a low velocity and continuously observe the local pressure. When the pressure reaches 30 mmHg, the embolic agent is stopped.

Step 2. Replace the new 4Fr catheter. Then water mixed with red dyes is injected into the target vessel at three different velocities using an automatic injection pump. These three velocities correspond to the injection velocities of the three curves in Fig. 3 at a pressure of 30 mmHg. Observe whether reflux occurs at different injection velocities.

Step 3. Replace the catheter and continue injecting the embolic agent. Repeat the second step when the pressure reaches 33, 35, 37, and 40 mmHg, respectively.

## Data Availability

All data generated or analyzed during this study are included in this published article.

## Abbreviations

DSA: Digital subtraction angiography
qDSA: Quantitative digital subtraction angiography
